# A qualitative study assessing the acceptability and adoption of implementing a results based financing intervention to improve maternal and neonatal health in Malawi

**DOI:** 10.1186/s12913-016-1652-7

**Published:** 2016-08-17

**Authors:** Danielle J. Wilhelm, Stephan Brenner, Adamson S. Muula, Manuela De Allegri

**Affiliations:** 1 Institute of Public Health, Medical Faculty, University of Heidelberg, Heidelberg, Germany; 2 Department of Public Health, School of Public Health, University of Malawi, College of Medicine, Blantyre, Malawi

**Keywords:** Results based financing, Acceptability, Adoption, Maternal health, Implementation research

## Abstract

**Background:**

Results Based Financing (RBF) interventions have recently gained significant momentum, especially in sub-Saharan Africa. However, most of the research has focused on the evaluation of the impacts of this approach, providing little insight into how the contextual circumstances surrounding the implementation have contributed to its success or failure. This study aims to fill a void in the current literature on RBF by focusing explicitly on the process of implementing a RBF intervention rather than on its impact. Specifically, this study focuses on the acceptability and adoption of the RBF intervention’s implementation among local and international key stakeholders with the aim to inform further implementation.

**Methods:**

The Results Based Financing for Maternal and Neonatal Health (RBF4MNH) Initiative is currently being implemented in Malawi. Our study employed an exploratory cross-sectional qualitative design to explore the factors affecting the acceptability and adoption of the intervention’s implementation. Purposeful sampling techniques were used to identify each key stakeholder who participated in all or parts of the implementation process. In-depth interviews were conducted and analyzed using a deductive open coding approach. The final interpretation of the findings emerged through active discussion among the co-authors.

**Results:**

Despite encountering several challenges, such as delay in procurement of equipment and difficulties in arranging local bank accounts, all stakeholders responded positively to the RBF4MNH Initiative. Stakeholders’ acceptance of the RBF4MNH Initiative grew stronger over time as understanding of the intervention improved and was supported by early inclusion during the design and implementation process. In addition, stakeholders took on functions not directly incentivized by the intervention, suggesting that they turned adoption into actual ownership. All stakeholders raised concerns that the intervention may not be sustainable after its initial program phase would end, which contributed to hesitancy in fully accepting the intervention.

**Conclusions:**

Based on the results of this study, we recommend the inclusion of local stakeholders into the intervention’s implementation process at the earliest stages. We also recommend setting up continuous feedback mechanisms to tackle challenges encountered during the implementation process. The sustainability of the intervention and its incorporation into national budgets should be addressed from the earliest stages.

**Electronic supplementary material:**

The online version of this article (doi:10.1186/s12913-016-1652-7) contains supplementary material, which is available to authorized users.

## Background

Results-based financing (RBF) in health can be defined as “a cash payment or non-monetary transfer made to a national or sub-national government, manager, provider, payer or consumer of health services after predefined results have been attained and verified” [1]. RBF is meant to place the incentive on outputs and outcomes rather than inputs and can include supply-side and demand-side incentives. Supply-side incentives, such as performance-based financing (PBF), offer health care providers additional monetary or non-monetary incentives to deliver specific services or meet targets pre-defined in a contract [1]. Demand-side incentives, such as conditional cash transfers, offer service users incentives to comply with use of specific health services [2].

RBF interventions have recently gained significant momentum, especially in sub-Saharan Africa, with more than 20 countries implementing or scaling-up programs [3]. The initial results from interventions using performance incentives are encouraging, but incentives need to be carefully designed and implemented to ensure success and avoid unintended consequences [4]. However, most studies on PBF have focused on the evaluation of the impacts of this approach, providing little insight into how the specific contextual circumstances surrounding the implementation have contributed to its success or failure [5]. A Cochrane Review, looking at PBF and its effects on the delivery of health interventions in low and middle-income countries, noted that the implementation process is key to interpreting the results of a PBF program, but that the mechanisms used to implement an intervention were rarely clear [6]. Rwanda has been frequently cited to indicate the successes of PBF, but little is known regarding PBF implementation even in this setting [7]. The same is true for demand-side incentives where studies have paid little attention to implementation processes [2]. Thus, it is impossible to ascertain if and under what circumstances the observed successes can be replicated in other settings [5].

This study aims to fill a void in the current RBF literature by assessing the process of implementing a RBF intervention rather than on its impact. Specifically, this study focuses on the acceptability and adoption of the RBF intervention among local and international key stakeholders. By identifying opportunities and challenges encountered during the implementation process, our study may be useful in guiding further implementation of this intervention and other RBF programs [8].

## Methods

### Study setting

Our study took place in Malawi, a low-income country in Southern Africa, which has high maternal and child mortality rates. Similar to most countries in sub-Saharan Africa, Malawi is not on track to achieve Millennium Development Goal 5 to reduce maternal mortality, in spite of recent progress showing the Maternal Mortality Ratio steadily decreasing from 1120/100,000 in the year 2000 to a figure of 510/100,000 in 2013 [9]. Surveys performed by the Ministry of Health indicated that several factors contribute to high maternal and neonatal mortality, including low quality of care, poor staff attitude, inadequate supplies, and difficulty obtaining or paying for transport to a health facility [10]. In 2004, an Essential Health Package was implemented in Malawi which delivers basic health services free of charge, through tax revenues and donor funds, but inadequate quality and access to services persist [11–13]. In addition, Malawi relies on a large amount of donor support to finance its health sector, accounting for 66 % of total health spending in 2008–2009 [14].

In line with the Millenium Development Goals and national health strategy, the Results Based Financing for Maternal and Neonatal Health (RBF4MNH) Initiative is currently being implemented in Malawi to improve the quality and access to maternal and neonatal health services [15, 16]. The Reproductive Health Directorate of the Ministry of Health (MoH) is implementing the intervention with technical support from Options Consultancy Services Ltd., a UK-based consultancy firm. The German Development Bank (KfW) and the Government of Norway, represented by the Royal Norwegian Embassy (RNE), are providing financial support. The intervention combines supply-side incentives typical of PBF schemes with demand-side incentives in the form of conditional cash transfers. The supply-side incentives are provided based on quality-performance contracts between district and local health facility teams and the Ministry of Health and include performance indicators consistent with emergency obstetric care standards (Table 1). The demand-side incentives consist of cash reimbursements to pregnant women to compensate for transport to a health facility for delivery and stay for 48 h after delivery (Table 2). Before and during the intervention, direct investments were made to upgrade buildings and provide equipment to guarantee minimum standards needed to provide quality maternal and neonatal health care services. The intervention is being implemented in the four districts of Balaka, Ntcheu, Dedza, and Mchinji. After months of preparation, the supply-side component began in April 2013 with verification and reward cycles occurring every 6 months. The demand-side component launched in different districts between November 2013 and June 2014. Data for this study was collected in June 2014, shortly after providers had received the second round of incentive payments.

**Table 1:** Supply Side Initiatives

Beneficiary	Indicators
Health Facility	1. Number of facility-based deliveries 2. Number of maternal and newborn deaths audits 3. Number of women tested for HIV if unknown status and treatment if indicated 4. Number and quality of Health Information Management Systems (HMIS) reports completed 5. Quality of stock cards for medications filled and submitted 6. Quality of RBF4MNH Initiative specific reports submitted 7. Number of completely filled partographs 8. Routine use of uterotonic in 3rd stage of labor 9. Routine use of magnesium sulfate for pre-eclampsia 10. Number of patient satisfaction surveys filled out for each quarter by women who delivered in facility 11. Routine administration of vitamin A to all newborns 12. Complete report on broken maternity equipment to District Health Officer 13. Routine use of infection prevention and quality checklist each month
District Health Management Team	1. Number of facility-based deliveries across districts 2. One-month supply of essential drugs and commodities available at all facilities in district 3. All essential equipment available in operating condition in participating facilities 4. Quality of Health Management Information Systems (HMIS) reports submitted to central office

**Table 2:** Demand Side Initiatives

Beneficiary	Indicators
Pregnant Women	Utilization of facility-based services at time of delivery
Women who delivered	Extension of facility stay to at least 48 h after delivery

### Conceptual framework and research design

Our study focused specifically on acceptability and adoption of the RBF4MNH Initiative among key stakeholders during its early implementation stages. Acceptability and adoption were selected as the focus of our analysis, since these two factors are critical to the initial stages of implementation of health interventions. We define acceptability as “the perception among implementation stakeholders that a given treatment, service, practice, or innovation is agreeable, palatable, or satisfactory” [17]. This includes any factors related to acceptability such as comfort or credibility of the intervention [18], which are particularly important during an intervention’s initial phase. We define adoption as “the intention, initial decision, or action to employ an innovation or evidence-based practice” [17]. Adoption refers to the uptake or intention to try a new intervention among stakeholders [18]. In addition, the sustainability of an intervention influences a stakeholder’s decision to fully invest in an intervention. These definitions of acceptability and adoption, along with the intervention’s design and sustainability, were used as a conceptual framework to assess the RBF4MNH Initiative implementation process.

Since the RBF4MNH Initiative is the first health intervention in Malawi employing RBF, an exploratory cross-sectional qualitative design was selected to explore possible factors affecting the implementation and to allow for a better understanding of key issues encountered during this process. This study examined the implementation process from the design phase beginning in November 2011 through part of the initial implementation phase from April 2013 to June 2014.

### Sampling

The authors were part of an independent research team set to evaluate the impact of the RBF4MNH Initiative on service use and quality of healthcare services [8]. Within the framework of this independent evaluation, the authors established frequent contact and information exchange with all concerned policy and implementation stakeholders. It is due to this constant exchange that the study team could identify all relevant stakeholders to be interviewed. The final study population consisted of 24 individuals, purposely selected to represent the totality of key policy and implementing stakeholders in the country. The sample included three representatives of the external project funders (one German Development Bank (KfW) technical expert, one Royal Norwegian Embassy (RNE) local health officer and deputy ambassador) and five central-level MoH representatives (the director of the Reproductive Health Directorate, the director of Planning and Policy, the former and current directors of the Sector Wide Approach Program (SWAp), and the zonal supervisor for the project area) who were responsible for aligning the intervention with national health priorities. Seven key representatives were selected from the implementation team, which included the Reproductive Health Directorate’s Chief Health officer, three external and three local technical consultants hired through Options Consultancy Services, Ltd. In addition, eight representatives from the District Health Management Teams (DHMT) were interviewed consisting of District Health Officers and District Nursing Officers in each of the four districts, who were actively involved in the implementation process and also direct beneficiaries of the rewards. Lastly, an interview was conducted with the local Health Program Director of the German Malawian Health Program (Deutsche Gesellschaft für Internationale Zusammenarbeit or GIZ) who provided additional technical support.

### Data collection

Data was collected through individual interviews with all respondents. Interviews were conducted directly by the first author either in person or via Skype. A total of seven semi-structured interview guides, each one specifically tailored to a different respondent group, were used as an aid during interviews (See Additional file 1). The interview guides were developed by the first author with the support of the co-authors and explored relevant factors related to acceptability and adoption of the RBF4MNH Initiative during the design and early implementation phases. Questions addressed the overall concepts of acceptability and adoption and specific probes investigated the motivation behind the initial responses. Interview guides were revised as the interviews proceeded to accommodate any additional emerging theme. All interviews were digitally-recorded. Written informed consent was obtained from the respondents prior to each interview. Each respondent was instructed that interviews could be declined or stopped at any time without consequences. Anonymity was provided to the greatest extent possible by referencing quotes to a particular stakeholder group instead of an individual’s specific position.

### Data analysis

The first author verbatim transcribed all digital interview recordings was responsible for the initial coding and analysis with support from the last author. Analysis began by organizing the transcribed material into meaningful units using a deductive open coding approach, with codes emerging as reading of the text progressed [19]. Once the initial coding was completed, the first and last author engaged in an iterative process to further organize coded text into overarching themes and to elucidate relationships between themes. The final interpretation of the findings, as presented in this manuscript, emerged through active discussion among the co-authors. Analysis was completed with the support of the software Nvivo.

## Results

We present findings according to the four conceptual elements at the core of our explorative study: the intervention’s introduction and design, acceptability, adoption and sustainability. Results are illustrated using direct quotations from the respondents.

### RBF4MNH initiative introduction and design

Introduction to the RBF4MMH Initiative varied depending on the role and position of the stakeholder interviewed. All of the central-level MoH respondents present at the start of the intervention, including Reproductive Health Directorate and SWAp members, were introduced to the RBF4MNH Initiative during its design phase. They stated that the German and Norwegian funding agencies initially promoted the idea of using development assistance for introducing RBF to Malawi. In turn, a feasibility study was commissioned to assess the potential relevance of introducing such an approach in Malawi. The stakeholders involved in the initial development of the RBF4MNH design described this process as a close collaboration of MoH members, funding agencies, and consultants. MoH respondents recounted that they were actively involved in contributing ideas during this initial phase, resulting in multiple changes to the rough design to meet everyone’s expectations.

*“It’s the Norwegians and the Germans who approached us with the money, then people really worked together to say what initiative we could do.” – MoH central-level member*

*“I was involved from the design stage up to the implementation. So whatever decision was made along the way I think I was an important part of it.” – MoH central-level member*

The District Health Management Teams (DHMT) were introduced early in implementation to assist with the identification of health facilities to participate in the intervention. However, approximately half of the interviewed DHMT members did not come into their position until after the facilities were already selected. DHMT members stated the primary method of introduction to the intervention was through group orientations targeting staff at each district hospital. These orientation meetings, which explained the supply and demand-side incentives of the RBF4MNH Initiative, were followed by additional trainings to reinforce these concepts. DHMT members recounted that the introduction to the intervention was performed either by consultants from Options Consultancy Services, Ltd., the Reproductive Health Directorate, or a combination of these two groups.

*“This orientation was done by the Ministry of Health government of Malawi, Reproductive Health Unit, in liaison with the results based financing personnel.” – DHMT member*

*“There were lots of meetings introducing the program where we were oriented as management. And where we had an input of what is supposed to happen and the choice of health centers.” – DHMT member*

The selection of performance indicators and targets on which to pay incentives, including the nature and amount of the financial rewards, represented the core of the intervention’s design. Eight respondents indicated that performance targets were based on existing maternal and neonatal health indicators used by the MoH. This process involved collaboration between the Reproductive Health Directorate, external and local consultants. As the implementation progressed, the Reproductive Health Directorate felt the indicators may be too ambitious, as one had to attain 100 % of the target or the beneficiary would not receive any of the reward. DHMT members stated that they had little involvement in the initial selection of indicators, but were allowed to give input into possible changes to the indicators and performance targets for the second phase of implementation. Despite not being directly involved in this process, all but one of the DHMT members were satisfied with the content of the indicators, because it was familiar to them. However, three DHMT members felt there were too many indicators or they did not have all the resources, such as medicine or equipment, necessary to achieve the performance targets.

*“They just used the indicators that the Ministry of Health is using. So it did not give a problem or any suspicion. We welcome the indicators.” – DHMT member*

*“The indicator will say you need to have this but you don’t have full control. So that’s another challenge (…) one faulty system will affect all the other indicators.” – DHMT member*

Amount and purpose of both supply-side rewards and demand-side payments were defined by the implementation team, with additional input from DHMT members concerning the distribution of supply-side financial rewards among beneficiaries at district and facility levels. Regarding the demand-side component, six DHMT members and the Reproductive Health Directorate approved of the cash transfers reimbursing women for travel expenses to health facilities, since they perceived that lowering the financial barrier could encourage more women to deliver at a facility. Despite approval of this aspect, half of the DHMT members and all but one central-level MoH member feared that the demand-side payments would encourage women to get pregnant for the purpose of receiving cash reimbursements. Therefore, respondents wished to see family planning strategies included in the demand-side component, such as giving reimbursements only to women who have waited 2 years since their last pregnancy to conceive again. Concerning the supply-side component, all central and district level MoH respondents considered performance incentives as a positive motivator for health worker performance. However, three DHMT members commented that the monetary value was not the most important aspect to them, but rather the public recognition themselves and their staff could receive.

*“I would say let’s emphasize more on family planning and take out the indicator of more deliveries every time (…) and let’s make sure we reward women who are compliant on family planning issues as well. I know the program is not aimed at increasing the number of pregnancies, but the interpretation in the community might be different if we don’t attach the family planning element.” – DHMT member*

*“But if we say that this facility has done well, then everybody is talking about it. Then they will get a t-shirt or a certificate. Then they will say, this district has done well. They have received that. And that would be more sustainable than money.” –DHMT member*

### Acceptability

The implementation team felt some central-level MoH members were initially reluctant to support the intervention fully. This reluctance was attributed to limited experience with RBF in combination with a rather general wait-and-see attitude. However, this reluctance changed rapidly over time, especially after the first rewards cycle when it became more obvious to stakeholders how the intervention was intended to work. The MoH members then realized the RBF4MNH Initiative could produce improved maternal health results. Specifically, the external consultants’ perceived that the general acceptability of the intervention grew stronger among local stakeholders once RBF’s potential in improving maternal care services provision was recognized.

*“After the first rewards cycle (…) there was this change [in MoH members] from observing to participation. And I think now within 2014, there’s a strong move towards ownership.” –external consultant*

The District Health Management Team response to the RBF4MNH Initiative was mostly positive. They attributed several changes to the intervention, including an increase in staff morale, enhanced teamwork, and an improvement in maternal and neonatal health services. Initially, three of the DHMT members stated they had concerns regarding the increased workload admitting this was a challenge at times. However, all but one DHMT member acknowledged the workload was manageable and still made progress in achieving the targets. Six DHMT members reported that their support for the intervention grew once they gained better understanding of how RBF worked. More than half of the DHMT members also viewed the support they received through the supervisory functions of the Reproductive Health Directorate as a key success factor in the implementation process.

*“The program at this point in time means a great improvement in the maternal-neonatal health indicators. And also it means boosting of staff morale (…). So it is a success to me.” –DHMT member*

All stakeholders stated that the RBF4MNH Initiative was a beneficial intervention despite some initial doubts and challenges. Central level MoH members and donors stated that they had already observed positive results such as increased staff motivation, cleaner facilities, and improvements in achieving performance targets from first to second round. Several DHMT members described their contribution to the intervention’s emerging design and appreciated that the implementation team considered their inputs. One DHMT member recognized this flexibility as an important factor in why this intervention was preferred over others where it was more difficult to initiate change.

*“I like the fact that it’s flexible. It’s not like they say this is what we have put in place and you have to follow it whether you like it or not. When we make an input they consider it. That’s what I like about it.” – DHMT member*

### Adoption

The MoH, and particularly the Reproductive Health Directorate, felt actively involved in guiding the implementation process from the earliest stages of the RBF4MNH Initiative. One consultant explained it took time for some central-level MoH members to be fully engaged in the intervention and suspected that this was due to previous health interventions in Malawi that did not allow for a more active involvement. Stakeholders noted that the Reproductive Health Directorate and consultants interacted closely with each other and even worked in the same office space, which allowed them to collaborate throughout the implementation process. All respondents felt involving stakeholders at administrative, local, district, and national levels in the early stages was important to the implementation process. After the design phase, the implementation team continued to work together to involve numerous other stakeholders including District Commissioners, community and traditional leaders, and hospital maintainers. This allowed the implementation team to address challenges and to make needed changes during the implementation process.

*“We always invited the District Commissioner and one other member from the local government so that we work together (…) we also involved the health education unit in the district, so that they could assist us in the sensitization of the districts about our program in the community.” – Reproductive Health Directorate*

All stakeholders who were directly involved in the implementation process described challenges that required adjustments to the intervention’s original design. Two challenges frequently discussed included the initial procurement of basic equipment and the set-up of financial structures within the RBF4MNH Initiative. All DHMT and implementation team members noted some delays in receiving essential equipment during implementation, which created difficulties for facilities to achieve certain targets. One consultant explained that these delays in equipment and supply procurement were overcome by using the intervention’s start-up portion of funds allocated to the facilities, which was corroborated by DHMT members. Although most of these start-up funds were used to purchase smaller items such as medications and curtains for the wards, the DHMT members reported that this created a better environment for the patients and assisted in attaining targets. In addition, guaranteeing the transfer of financial rewards to facility staff and cash reimbursements to enrolled women was challenging. The local accountant recalled that the establishment of structures regulating financial flow between the central RBF office and the facilities was a complicated process. The implementation team initiated setting up bank accounts at the district and facility levels, but MoH members were resistant to this idea for fear of funds being mismanaged at the facility level due to lack of financial expertise. Therefore, bank accounts could only be established at the district level, from which funds were disbursed to the facilities. According to the accountant, this lengthy process forced the implementation team to shoulder some of the financial responsibility until the process of transferring funds to facilities was clearly understood at the district level.

*“We felt that the people that are at the health centers are not trained to keep monies. Were we not going to misappropriate funds that the donors have faithfully given to us to improve the lives of the Malawians?” – central-level MoH member*

*“It already was a problem to have the specific [bank] account at the district level and it took a very long time. So there are some steps in this procedure where the money gets stuck. And then you run the risk that the facilities run out of money.” – external consultant*

All stakeholders brought up shortage of hospital staff in Malawi as another critical challenge. DHMT members stated this caused difficulties meeting certain performance targets, especially when it came to additional documentation necessary for reporting and verifying achievement of the targets. Two DHMT members indicated that the increased workload was partly overcome by the intervention requiring participating facilities to have a minimum number of staff. This requirement, however, created an additional challenge as staff members needed to be moved from non-intervention to intervention facilities. This issue was exacerbated by the high staff turnover rate existing at local and national levels, which stakeholders cited as a factor affecting implementation as orientation of new staff took time. A local consultant explained that the implementation team involved the entire DHMT to ensure someone at the district level would always be familiar with the intervention and felt this was an effective coping strategy.

*“With the program we knew we would have this workload. Now knowing that we are already short staffed and then with that workload, we are struggling to meet the indicators.” – DHMT member*

All but one DHMT member recounted how quarterly district review meetings assisted in communication and addressing challenges. When asked about the district review meetings, every DHMT member responded the meetings were useful to share information and suggestions, which allowed the implementation team to make necessary changes to the intervention. Supervision and feedback mechanisms were used at district and facility levels as another way to discuss gaps and strategies to improve performance. DHMTs stated they used a variety of methods to assist the facilities, which included coordination meetings, identifying problems in achieving the indicators, and, in one case, coming up with specific action plans after looking at patient exit questionnaires to identify causes of client complaints.

*“[District review meetings] are really good, because we discuss issues on the ground affecting implementation of the Initiative. As we share information and suggestions are made, actions plans are made, and we implement and see a change.” – DHMT member*

Despite challenges, adoption of the RBF concept began to occur not only at the district level, but also at the highest levels of the MoH and government. A consultant explained that it took DHMTs several months to understand the concepts and to be trained in the additional tasks required for the RBF intervention, but DHMTs are now translating this knowledge into action to achieve the targets through better supervision and management. According to six of the DHMT members, the Reproductive Health Directorate has been seen as implementing the intervention through frequent visits to all participating facilities and providing supporting as challenges arose. However, one DHMT member stated that consultants from Options Consultancy Services, Ltd., rather than the Reproductive Health Directorate, were more involved in implementing and supporting the DHMT. In regards to adopting the intervention among higher levels of government, the implementation team commented that the MoH and the Directorate of Planning and Policy has considered including RBF in future health strategies and plans.

*“They [MoH] are talking about RBF in their plans. When they are going out for supervision…they can also see the positive affect of it already. So yes, there is ownership now.” –Funding agency*

### Sustainability

All but one stakeholder spontaneously commented on sustainability, which was discussed throughout the design and implementation processes. One consultant stated that this was the most frequently cited concern among all stakeholders. One respondent specifically noted how sustainability of the intervention affected early willingness to get fully involved in the implementation process as local stakeholders questioned if the intervention could continue beyond its initial phase. Every Malawian stakeholder was concerned with what would happen once external funders stopped supporting the intervention. All DHMT members would like the intervention to continue and to expand to other facilities. Half of the stakeholders reported the presence of political will to sustain the RBF4MNH Initiative. All but one respondent felt that the RBF4MNH Initiative could be sustained from a technical perspective without external support, but all stakeholders doubted that the Malawian government alone would be able to sustain it financially. Consultants and MoH members pointed to the need to integrate RBF into the national budget in order for it to be sustained.

*“If it doesn’t continue, it will demoralize people. The commitment will go back to continue delivering in the community. So that is our biggest fear.” – DHMT member*

*“The issue was at the end of the day is how would we integrate the finances into the budget, into the general budget. How would we do it using the regular budget?” – MoH member*

## Discussion

Despite encountering several challenges, all stakeholders responded positively to the implementation of the RBF4MNH Initiative. Although the intervention was initially promoted by foreign funding agencies, local stakeholders quickly accepted and adopted it. This is likely due to fact that local stakeholders saw the intervention as being legitimate and trusted the implementation team, which consisted of representatives from the Reproductive Health Directorate and local Malawian consultants in addition to external consultants. By involving key stakeholders such as central-level MoH members, DHMTs, hospital and administrative staff, district government leaders, as well as community leaders during initial design and planning phases, the implementation team was able to promote acceptance and overcome resistance that can often occur in a more typical top-down approach [20]. As noted in other RBF interventions, this buy-in from the stakeholders is important as their involvement and decisions during the design process will affect them and the intervention’s implementation [4, 21]. Through inputs from district authorities and MoH members, the implementation team could better align the intervention with the actual country needs leading to greater acceptance among local stakeholders. The involvement of local actors and allowing the Ministry of Health to make critical decisions to guide the process proved to be important throughout implementation in developing ownership. In addition, the local government is now considering RBF in future health plans demonstrating political commitment, which has been identified as a key success factor in several RBF interventions [20].

During a RBF design phase, it is critical that performance indicators, targets, and incentives are relevant to the context [20]. In an Ugandan PBF intervention, for example, targets were hastily chosen, contributing to many challenges during implementation, since indicators did not align with the goals of the health facilities [22]. The RBF4MNH Initiative managed to avoid such pitfalls by aligning their objectives with those previously established by the national health plans. Hospital staff more easily accepted the indicators, since they were congruent with recognized clinical guidelines. However, Reproductive Health Directorate and DHMT members noted that the targets may have been too ambitious initially and advocated for changes to be made to the indicators. Despite this, all stakeholders commented on the supply-side incentives as a positive motivator. Moreover, DHMT members stated that it was nice to have someone appreciate their work, which has also been seen as an important factor among health workers participating in RBF interventions [23].

Regarding the demand-site incentives, many stakeholders were not entirely supportive of the conditional cash transfers. Although stakeholders recognized the value of cash transfers allowing pregnant women to be reimbursed for transport costs to a facility, there was a strong sentiment that these incentives may encourage women to get pregnant. This was one of the main criticisms to the RBF4MNH design, especially in regards to Malawi’s high fertility rate, and Malawian stakeholders perceived that the conditional cash transfers were contradictory to their efforts in promoting family planning [12]. Other demand-side interventions have found it challenging to strike this balance between financial support of pregnant women and respect towards family planning efforts [24].

Our findings indicate that stakeholders’ acceptance of the RBF4MNH Initiative grew stronger over time as understanding of the intervention improved. As reported in several World Bank-funded RBF interventions, the mindset shift from an input-based to a results-based financing approach takes time [20]. The experience reported by our respondents is therefore not unexpected as acceptability of any intervention is likely to increase with the knowledge of that intervention [17]. However, moving from acceptance to adoption can be a challenging process, since the latter requires further sustained efforts by translating this knowledge into action [18]. Our findings demonstrate that a number of stakeholders waited for the first payment cycle to be completed before getting actively involved. This suggests that, without being overtly resistant to the intervention, many key stakeholders waited to witness its benefits before committing to its implementation.

During the RBF4MNH Initiative’s implementation, stakeholders faced some challenges requiring changes to be applied to the intervention’s original design. This is common in complex interventions to ensure that the needs of various stakeholders are met [25]. The delay in receiving essential equipment, for instance, proved to be an important challenge as DHMTs felt this affected their ability to achieve certain targets. These obstacles risk demoralizing providers as they may feel unfairly deprived of financial rewards due to factors beyond their control [26]. Although a delay in equipment was frustrating, DHMTs used start-up funds to procure needed items, such as medicines and basic equipment, and were able to meet targets. Additionally, the implementation team encountered difficulties in transferring money to all beneficiaries since establishing facility bank accounts was administratively impossible. Highly centralized systems, which weaken local authority, are challenging to RBF interventions as their financing structures need to rely on the ability to mobilize and manage resources at the local rather than central level [5].

The shortage of hospital staff, which is a well-known concern in Malawi [27], led to difficulties in completing RBF-related documentation, even though facility personnel was aware that these tasks were necessary to achieve performance targets and receive the related payment. In a Rwandan PBF intervention, health workers felt this type of administrative burden to achieve targets absorbed more of their time and effort than the performance of the actual health service [23]. Some stakeholders indicated that high staff turnover at central and district levels also delayed the implementation process. In PBF projects in Ecuador and Bolivia, high staff turnover also caused a major obstacle in coordinating responsibilities of stakeholders within the interventions [20].

Quarterly district review meetings were included in the RBF4MNH Initiative’s design to ensure concerns were addressed during the implementation process. Arranging such a feedback mechanism became an important factor in the success or failure of other PBF interventions [22], and should be established at local, district, and central levels in order to discuss and inform all key stakeholders on the challenges and progress being made [20]. Some DHMT members even took it upon themselves to develop the most appropriate mechanism to give feedback to their facilities. The fact that stakeholders took on functions not directly incentivized by the intervention, suggests that they turned adoption into actual ownership.

Although assessing sustainability was not our original intention, our findings indicated that this issue greatly affected the acceptability and adoption of the intervention. All stakeholders raised concerns regarding sustainability, without specifically questioned about this matter during the interviews. Fear that the RBF4MNH Initiative may not be sustainable after its initial program phase has ended, contributed to initial hesitancy in fully accepting the intervention. Ensuring sustainability is a challenge to RBF interventions, but few guidelines or solutions have been discussed [20]. Despite these concerns, after having experienced the intervention to increase providers’ motivation and quality of service provision, all stakeholders wished to see the RBF4MNH Initiative continue. Still, sustainability issues raise additional concerns about the potential negative effect on providers’ motivation should the incentives be discontinued [23].

## Conclusion

These findings are new to the field of implementation research with no known previous studies examining the acceptability and adoption of the implementation of an RBF intervention. Based on the results of this study, we recommend the active involvement of stakeholders during the intervention’s implementation process and encourage input from every stakeholder level during the design and implementation phases. We also learned from the results of this study the importance of continuous feedback mechanisms to tackle challenges encountered during the implementation process. In addition, implementation teams should consider the needs and wishes of the local stakeholders when designing indicators and incentives. Finally, the sustainability of the intervention and its incorporation into national budgets should be addressed from the earliest stages. Although certain findings are specific to the RBF4MNH Initiative, these findings still lend insight into project implementation aspects relevant to other low-income countries considering implementation of RBF schemes.

## Additional file

Additional file 1: Interview Guides. All interview guides used during data collection for each group of participants. (PDF 109 kb)

## Abbreviations

DHMT: District health management team
MoH: Ministry of health
PBF: Performance-based financing
RBF: Results-based financing
RBF4MNH: Results-based financing for maternal and neonatal health
SWAp: Sector wide approach program
