# Supplementary material for: A qualitative study assessing the acceptability and adoption of implementing a results based financing intervention to improve maternal and neonatal health in Malawi
Source: BMC Health Serv Res. 2016 Aug 17;16:398. doi: 10.1186/s12913-016-1652-7 (PMC4989348; doi:10.1186/s12913-016-1652-7)
Supplement: Additional file 1: — Interview Guides. All interview guides used during data collection for each group of participants. (PDF 109 kb) [file 12913_2016_1652_MOESM1_ESM.pdf]

Additional File 1: Interview Guides

In-Depth Interview Guide

Assessing the Acceptability and Adoption of Implementing a Results Based Financing Project to Improve Maternal and Newborn Health in Malawi

**Archival Number**			**Affiliation**			
**Name of Moderator**						
**Date**			DD	MM	YYYY	
**Start Time**			**End Time**			

Introduction:

I am a Masters student at Heidelberg University in Germany. The aim of this study is to learn about the experience, challenges, and successes of the implementation of Results Based Financing for Maternal and Neonatal Health Initiative (RBF4MNH) in Malawi. This study is part of the larger impact evaluation of the RBF4MNH project funded by the German Promotional Bank and Norway and run by the Ministry of Health with technical support by Options.

I have asked to interview you, because you are a key person with knowledge and insight regarding the implementation of the RBF4MNH project and I am interested in learning your opinions and experiences. I would like for you to provide your own personal experiences and thoughts regarding the RBF4MNH project, the acceptability of the project, responses taken in regards to implementation, and the factors affecting implementation.

I have reviewed the procedures for the interview during the consent process. Do you have any further questions before we begin?

Ministry of Health/Reproductive Health Directorate Interview Guide

1. Please describe your role and position in the Ministry of Health.
   Probes: What is your formal position?
   What are your duties and responsibilities?
   What do you do in practice?

2. What is your role and position specifically within the RBF4MNH program?
   Probes: What is your formal position?
   What are your responsibilities?
   What do you do in practice?

3. What was your knowledge of RBF prior to the initiation of this program?
   Did you have any opinions about RBF programs (in general)?

4. How did you first hear about the RBF4MNH program?
   How were you introduced to the program?
   How long have you been working within the RNF4MNH program?

5. How was the design and concept of the RBF4MNH program explained to you?
   How were your specific role and responsibilities explained to you?
   What level of input did you have in defining your responsibilities?

6. What initial decisions needed to be made in order to employ this new intervention in Malawi?
   Probes: Indicators? Incentives? Location?
   Who made these decisions?
   Probes: MOH, Options, collaboration?
   How were these decisions made?
   Why were these decisions made (as they were)?

7. How were the districts chosen that would be involved in the project?
   Probe: Who chose these districts? Why were these districts chosen?
   How were the facilities chosen within the district?
   Probe: Who chose these facilities? Why were these facilities chosen?

8. What actions or strategies were taken to assist with implementation of RBF4MNH in the participating districts?
   Who implemented these strategies?
   Probes: MoH, RHU, OPTIONS, combination?
   How were these strategies implemented?
   Probes: In every district? Facility?

9. What were some of the factors that influenced your responses or actions during the initial phase of implementation of the RBF4MNH program?
   Probe: What were the reasons why certain actions were taken?

10. How has your overall experience been with the implementation of RBF during this initial phase?

What have been the successes?

Probes: Who was responsible for these successes? How were these successes achieved?

What challenges or obstacles did you face?

Probes: By whom? How did you overcome these obstacles?

11. Did those involved in the project abide by the plan that was made for implementation?

Probe: If not, why not?

What changes needed to be made?

Probe: How were these changes implemented? Why were they implemented?

12. What were any surprises or aspects of implementing RBF4MNH that you were not expecting?

Why was it a surprise? How did you respond to unexpected events?

13. How, in your opinion, has been the general response of (i) the Ministry of Health and (ii) the government to the RBF4MNH program?

Probe: Response of RHU?

What was the level of comfort with this program?

Was your department agreeable to this program?

Were there objections to this program?

14. How, in your opinion, has been the general response of (i) KfW and RNE and (ii) OPTIONS to the RBF4MNH program?

In your opinion, what is the level of support towards your department?

How responsive have they been to issues, if any, brought up by your department?

15. How, in your opinion, has been the general response of district health officers and providers to the RBF4MNH program?

Were there any differences across districts? Facilities?

In your opinion, where were the reasons for these differences?

16. What would you recommend to do differently if you were to restart today with the experience you have gathered in the meantime?

17. What would you do differently if you had to implement RBF in different regions?

18. Is there anything further that you would like to add about the topics that we have spoken about today?

District Health Management Team Interview Guide

1. Please describe your role and position in the District Health Management Team.
   Probes: What is your formal position?
   What are your duties and responsibilities?
   What do you do in practice?

2. What is your role and position specifically within the RBF4MNH program?
   Probes: What is your formal position?
   What are your responsibilities?
   What do you do in practice?

3. What was your knowledge of RBF prior to the initiation of this program?
   Did you have any opinions about RBF programs (in general)?

4. How did you first hear about the RBF4MNH program?
   How were you introduced to the program?
   How long have you been working within the RNF4MNH program?

5. How was the concept of the RBF4MNH program explained to you?
   Were you involved in the feasibility study?
   Probe: If yes, how were you involved?
   What level of input did you have in the design of the program?
   Probe: What ideas did you contribute to the program? How did the implementation team receive or respond to your ideas? Did you have any objections to the program or parts of the program?

6. How were your specific role and responsibilities determined within the program?
   Probe: What level of input did you have in defining your own responsibilities in regards to the program? What were your objections, if any?

7. What initial decisions needed to be made in order to start this new intervention in Malawi?
   Probes: Indicators? Incentives? Location?
   Who made these decisions? How much were you involved in these decisions?
   Probes: MOH, Options, collaboration? How were these decisions made?
   Why were these decisions made (as they were)?
   Probes: Did you have any objections to these decisions? If so, why?

8. What actions or strategies did you take to assist with implementation of RBF4MNH your district?
   What was the responsibility of the DHMT in implementing these strategies? Did others assist in implementing these strategies?
   Probes: MoH, RHU, OPTIONS, combination?
   How were these strategies implemented?

Probes: In every facility?

9. What were some of the factors that influenced your responses or actions during the initial phases of the RBF4MNH program?

Probe: What were the reasons why you took certain actions?
Responses or attitude of providers? Resources available?

10. How has your overall experience been with the implementation of RBF during this initial phase?

What have been the successes?

Probes: Who was responsible for these successes? How were these successes achieved?

What challenges or obstacles did you face?

Probes: By whom? How did you overcome these obstacles?

11. Were you always able to do what the program asked you to do? Were you able to influence what you were asked to do?

Probe: If not, why not?

What changes needed to be made? How much input did you have in these changes?

Probe: How did you implement these changes? Why were they implemented?

How did you feel about making these changes in regards to how you work?

Probe: Increased workload? More supervision?

12. Were there any surprises or aspects during the initial phases of implementation of RBF4MNH that you were not expecting?

Why was it a surprise? How did you respond to unexpected events?

13. How much support do you feel you receive from the Ministry of Health in regards to the RBF4MNH program?

Probe: Support of RHU?

How responsive have they been to issues, if any, brought up by you or the DHMT?

14. How, in your opinion, has been the general response of OPTIONS to the DHMTs in regards to the RBF4MNH program?

What is the level of support OPTIONS have given towards the DHMT?
How responsive have they been to issues, if any, brought up by the DHMT?

15. Did you have enough contact with the other DHMTs to see how they responded to the program?

If so, how did they react to the program? What changes have you seen them make due to this program?

Probes: Were there any differences across districts? Facilities?
In your opinion, what were the reasons for these differences?

16. How, in your opinion, has been the general response of the providers in your district to the RBF4MNH program?

Were they agreeable to the plan? How did they feel about the contracts? How did they feel about what they were asked to do? Feelings about the incentives?

Probe: Were there any specific objections to the plan? If so, what were these objections? How were their concerns addressed?

How did this program affect the relationship between those included and those excluded from the intervention? How did it affect your work?

17. At this point, what are your feelings in regards to the RBF4MNH program?

Probe: What are your perceptions about the program?

How do you feel in regards to your level of participation in this program?

18. What would you recommend to do differently if you were to restart today with the experience you have gathered in the meantime?

19. What would you do differently if you had to assist with implementation of RBF in different regions?

20. Is there anything further that you would like to add about the topics that we have spoken about today?

Royal Norwegian Embassy Interview Guide

1. Please describe your role and position in the Royal Norwegian Embassy?

Probes: What is your formal position?

What are your duties and responsibilities?

Do you perform other duties outside of your formal position?

2. What is your role and position specifically in regards to the RBF4MNH program?

Probes: What is your formal position?

What are your responsibilities?

Do you perform additional duties outside of the scope of your formal position/title?

3. How did you first hear about the RBF4MNH program?

How were you introduced to the program?

How long have you been working within the RNF4MNH program?

4. How did RNE come up with the idea of supporting a project such as RBF4MNH?

Probe: Why was this particular project supported?

Who initiated the idea for this project?

4. Why was results-based financing chosen as the method to improve maternal/neonatal health for this project?

Probe: How was RBF chosen for this project?

Who initiated this idea?

5. Why was Malawi chosen for this project?

Probe: How was the decision made to choose Malawi?

Who chose Malawi for this project?

4. Can you describe how the design and concept of the RBF4MNH program developed?

Who took charge of the design? To what extent was RNE involved in shaping ideas?

Probe: Were you directly involved in this process or parts of the process?

Did you run into any barriers while developing the RBF4MNH program?

If so, who was responsible? How did you overcome these barriers?

5. What do you know regarding the implementation of the program in Malawi?

Probe: Do you have any direct involvement? What is your level of involvement?

6. Do you know what initial decisions needed to be made in order to launch this new intervention in Malawi?

Probes: Incentives? Method of payment? Overall funding?

Who made these decisions?

Probes: KfW/RNE, MOH, Options, collaboration?

How were these decisions made? Why were these decisions made (as they were)?

7. Do you know how the districts were chosen that would be involved in the project?

Probe: Who chose these districts? Why were these districts chosen?

How were the facilities chosen within the district?

Probe: Who chose these facilities? Why were these facilities chosen?

8. In your opinion, how has the communication been between OPTIONS and RNE?

Probe: Any difficulties in communications? How were these overcome?

Was OPTIONS receptive to KfW/RNE contributing ideas?

How responsive was OPTIONS to new ideas that came from beyond their team?

9. In your opinion, how has the communication been between the MoH and RNE?

Probe: Any difficulties in communications? How were these overcome?

Was MoH receptive to RNE contributing ideas?

10. Do you know of any changes that needed to be made with the plan? What were the changes?

Probe: Who communicated these changes? How did they communicate those changes to you? Were the communications made in a timely fashion?

11. How has the communication been between KfW and RNE in regards to RBF4MNH?

Probe: Were there any difficulties in communication?
If so, how were these difficulties overcome?

12. How, in your opinion, has been the general response of (i) the Ministry of Health and (ii) the government to the RBF4MNH program?

Probe: Response of RHU?

Was the MoH/government agreeable to the program? If not, why not?
Were there specific objections to this program? If so, who raised these objections? Why were the objections raised? How were they addressed?

13. How, in your opinion, has been the general response of district health officers and providers to the RBF4MNH program?

Were there any differences across districts? Facilities?
In your opinion, what were the reasons for these differences?

14. What has been the local response by the donor and policy community to this program?

How has this program changed RNE's perception or standing locally as a donor?

Probe: Why do you think perceptions have changed the way that they have?

How has the program changed KfW's perception or standing locally as a donor?

15. What is your overall impression of the implementation of RBF4MNH during this initial phase?

Were there any delays in the implementation?

Probes: Do you know why these delays occurred?
Were you informed in time?

16. How satisfied or unsatisfied are you with the implementation of the program to this point?

How satisfied are you with the results of the implementation?
Would you recommend that RNE finance a project like this in the future?

Probe: Why or why not?

17. What were any surprises or aspects of RBF4MNH that you were not expecting?

Why was it a surprise? How did you respond to unexpected events?

18. What would you recommend to do differently if you were to restart today with the experience you have gathered in the meantime?

19. What would you do differently if you had to implement RBF in different regions?

20. Is there anything further that you would like to add about the topics that we have spoken about today?

German Development Bank (KfW) Interview Guide

1. Please describe your role and position in KfW?

Probes: What is your formal position?

What are your duties and responsibilities?

Do you perform other duties outside of your formal position?

2. Please describe your role and position specifically in regards to the RBF4MNH program.

Probes: What is your formal position?

What are your responsibilities?

Do you perform additional duties outside of the scope of your formal position/title?

3. How did KfW come up with the idea of supporting a project such as RBF4MNH?

Probe: Why was this particular project supported?

Who initiated the idea for this project?

4. How was results-based financing chosen as the method to improve maternal/neonatal health for this project?

Probe: Were other methods considered? Why RBF chosen?

Why was it combined with conditional cash transfers?

Who initiated these ideas?

5. How was the decision made to choose Malawi for this project?

Probe: Why was Malawi chosen for this project?

Who chose Malawi for this project?

4. Can you describe how the design and concept of the RBF4MNH program developed?

Did you run into any barriers while developing the RBF4MNH program?

If so, can you describe these barriers? How did you overcome these barriers?

Probe: Feasibility study? Tender? Decision on who would implement?

5. What do you know regarding the implementation of the program in Malawi?

Probe: What is your level of involvement?

6. What initial decisions needed to be made in order to employ this new intervention in Malawi?

Probes: Incentives? Method of payment? Overall funding?

Who made these decisions?
Probes: KfW/RNE, MOH, Options, collaboration?
How were these decisions made?
Why were these decisions made (as they were)?

7. How were the districts chosen that would be involved in the project?
Probe: Who chose these districts? Why were these districts chosen?
How were the facilities chosen within the district?
Probe: Who chose these facilities? Why were these facilities chosen?

8. How, in your opinion, has been the general response of (i) the Ministry of Health and (ii) the government to the RBF4MNH program?
Probe: Response of RHU?
Was the MoH/government agreeable to the program? If not, why not?
Were there specific objections to this program? If so, who raised these objections? Why were the objections raised? How were they addressed?
Probe: Why separate agreement for a steering committee?

9. How, in your opinion, has been the general response of district health management teams to the RBF4MNH program?
Were there any differences across districts? Facilities?
In your opinion, what were the reasons for these differences?

10. What is your overall impression of the implementation of RBF4MNH during this initial phase?
Were there any delays in the implementation?
Probes: Do you know why these delays occurred?
Were you informed in time?

11. Can you describe the communication between OPTIONS and KfW?
Probe: Any difficulties in communications? How were these handled?
Was OPTIONS receptive to KfW/RNE contributing ideas?
How responsive was OPTIONS to new ideas that came from beyond their team?

12. Did those implementing the project communicate changes that needed to be made with the plan?
Probe: How did they communicate those changes to you?
Were the communications made in a timely fashion?

13. Can you describe the communication between KfW and RNE in regards to RBF4MNH?
Probe: Were there any difficulties in communication?
If so, how were these difficulties handled?

14. How satisfied or unsatisfied are you with the implementation of the program to this point?

How satisfied are you with the results of the implementation?

Would you recommend that KfW finance a project like this in the future?

Probe: Why or why not?

15. What were any surprises or aspects of RBF4MNH that you were not expecting?

Why was it a surprise? How did you respond to unexpected events?

16. What would you recommend to do differently if you were to restart today with the experience you have gathered in the meantime?

17. What would you do differently if you had to implement RBF in different regions?

18. Is there anything further that you would like to add about the topics that we have spoken about today?

External Consultants (Options Consultancy Services) Interview Guide

1. Skip for consultants: Please describe your role and position in OPTIONS.

Probes: What is your formal position?

What are your duties and responsibilities?

What do you do in practice?

2. What is your role and position specifically within the RBF4MNH program?

Probes: What is your formal position?

What are your responsibilities?

What do you do in practice?

3. What was your knowledge of RBF prior to the initiation of this program?

Had you worked on RBF projects previously?

Did you have any opinions about RBF programs (in general)?

Were you already involved during the initial feasibility study or only became involved later? If so, what was your role during the feasibility study?

4. How did you first become involved or interested in the RBF4MNH program?

How long have you been working within the RNF4MNH program?

5. Do you recall how the design and concept of the RBF4MNH program developed?

What specific responsibilities did you have in the design and plan?

What level of input did you have in the design of the RBF4MNH program?

What level of input did other stakeholders (MoH, donors) have?

6. What initial decisions needed to be made in order to employ this new intervention in Malawi?

What was your role in mediating these decisions? How much was the MoH involved in this initial phase? Who specifically?

Probes: Indicators? Incentives? Location?

How were these decisions made?

Why were these decisions made (as they were)?

7. How were the districts chosen that would be involved in the project?

Probe: Who chose these districts? Why were these districts chosen?

How were the facilities chosen within the district?

Probe: Who chose these facilities? Why were these facilities chosen?

8. What actions or strategies were taken to assist with implementation of RBF4MNH in the participating districts?

Who implemented these strategies?

Probes: MoH, RHU, OPTIONS, combination?

How were these strategies implemented?

Probes: In every district? Facility?

9. How has your overall experience been with the implementation of RBF during this initial phase?

What have been the successes?

Probes: Who was responsible for these successes? How were these successes achieved?

What challenges or obstacles did you face?

Probes: By whom? How did you overcome these obstacles?

10. At this point, is the RBF4MNH program a reflection of what was originally envisioned?

What changes needed to be made along the way?

Probe: Why were these changes made? How were changes made from initial plan?

Were there any factors influencing the implementation?

Probe: Policy changes? Other RBF programs? Other health sector interventions in participating districts?

Did those involved in the project abide by the plan that was made for implementation?

Probe: If not, why not?

12. Were there any surprises or aspects of implementing RBF4MNH that you were not expecting?

Why was it a surprise? How did you respond to unexpected events?

13. How, in your opinion, has been the general response of (i) the Ministry of Health and (ii) the government to the RBF4MNH program?

Probe: Response of RHU?

Was the MoH/government agreeable to the program? If not, why not?

Were there specific objections to this program? If so, who raised these objections? Why were the objections raised? How were they addressed?

Probe: CHAM health facilities?

14. How, in your opinion, has been the general response of district health officers and providers to the RBF4MNH program?

Were there any differences across districts? Facilities?

In your opinion, what were the reasons for these differences?

Probe: Trainings? Meetings? QI circles?

15. How, in your opinion, has been the general response of (i)KfW and RNE (German Development Bank and Norway) and (ii) development partners to the RBF4MNH program?

What is the level of support towards the program?

How responsive have they been to issues, if any, brought up during implementation?

Probe: Steering committee? Investment in infrastructure?

What attention has the program received at national and international levels?

16. How, in your opinion, has been the general response of OPTIONS team members local to Malawi to the RBF4MNH program?

What is the level of support you received from local Malawi team members?

Did they have any objections to the program or parts of the program? If so, how were these addressed?

17. What would you recommend to do differently if you were to restart today with the experience you have gathered in the meantime?

18. What would you do differently if you had to implement RBF in different regions?

19. Is there anything further that you would like to add about the topics that we have spoken about today?

Local consultants (Options Consultancy Services) Interview Guide

1. Skip if consultant: Please describe your role and position with OPTIONS.

Probes: What is your formal position?

What are your duties and responsibilities?

What do you do in practice?

2. What is your role and position specifically within the RBF4MNH program?

Probes: What is your formal position?

What are your responsibilities?

What do you do in practice?

3. What was your knowledge of RBF prior to the initiation of this program?
   Had you worked on RBF projects previously?
   Did you have any opinions about RBF programs (in general)?

4. How did you first hear about the RBF4MNH program?
   How were you introduced to the program?
   How long have you been working within the RNF4MNH program?

5. Do you recall how the design and concept of the RBF4MNH program developed?
   What specific responsibilities did you have in the design and plan?
   What level of input did you have in the design of the RBF4MNH program?
   What level of input did other stakeholders (MoH, donors) have?

6. What initial decisions needed to be made in order to employ this new intervention in Malawi?
   What was your role in mediating these decisions? How much was the MoH involved in this initial phase? Who specifically?
      Probes: Indicators? Incentives? Location?
   Who made these decisions?
      Probes: MOH, Options, collaboration?
   How were these decisions made? Why were these decisions made (as they were)?

7. How were the districts chosen that would be involved in the project?
      Probe: Who chose these districts? Why were these districts chosen?
   How were the facilities chosen within the district?
      Probe: Who chose these facilities? Why were these facilities chosen?

8. What actions or strategies were taken to assist with implementation of RBF4MNH in the participating districts?
   Who implemented these strategies?
      Probes: MoH, RHU, OPTIONS, combination?
   How were these strategies implemented?
      Probes: In every district? Facility?

9. What were some of the factors that influenced your responses or actions during the initial phase of implementation of the RBF4MNH program?
      Probe: What were the reasons why certain actions were taken?

10. How has your overall experience been with the implementation of RBF during this initial phase?
   What have been the successes?
      Probes: Who was responsible for these successes? How were these successes achieved?

What challenges or obstacles did you face?

Probes: By whom? How did you overcome these obstacles?

11. Did those involved in the project abide by the plan that was made for implementation?

Probe: If not, why not?

What changes needed to be made?

Probe: How were these changes implemented? Why were they implemented?

12. What were any surprises or aspects of implementing RBF4MNH that you were not expecting?

Why was it a surprise? How did you respond to unexpected events?

13. How, in your opinion, has been the general response of (i) the Ministry of Health and (ii) the government to the RBF4MNH program?

Probe: Response of RHU?

Was the MoH/government agreeable to the program? If not, why not?
Were there specific objections to this program? If so, who raised these objections? Why were the objections raised? How were they addressed?

14. How, in your opinion, has been the general response of district health officers and providers to the RBF4MNH program?

Were there any differences across districts? Facilities?
In your opinion, what were the reasons for these differences?

15. How, in your opinion, has been the general response of (i)KfW and RNE (German Development Bank and Norway) and (ii) other development partners to the RBF4MNH program?

What is the level of support towards the program?
How responsive have they been to issues, if any, brought up during implementation?
What attention has the program received at national and international levels?

16. How, in your opinion, has been the general response of OPTIONS management to the local team members working for the RBF4MNH program?

What level of support for this program did you received from OPTIONS as a local team member?
Did you, as a local team member, have specific objections to the program or parts of the program? If so, how did OPTIONS address these concerns?

17. What would you recommend to do differently if you were to restart today with the experience you have gathered in the meantime?

18. What would you do differently if you had to implement RBF in different regions?

19. Is there anything further that you would like to add about the topics that we have spoken about today?

Deutsche Gesellschaft für Internationale Zusammenarbeit (GiZ) Interview Guide

1. Please describe your role and position with GIZ.
Probes: What is your formal position?
What are your duties and responsibilities?
What do you do in practice?

2. How did you first hear about the RBF4MNH program?
Probe: How were you introduced to the program?

3. How was the design and concept of the RBF4MNH program explained to you?
Probe: Who explained the RBF4MNH program to you?

4. What was your knowledge of RBF prior to the initiation of this program?
Did you have any opinions about RBF programs (in general)?

5. What is your contact with the RBF4MNH program?
Probes: Do you assist with the project?
If so, in what areas do you assist the project? How do you assist the project?
What is your level of interaction with the program?

6. Does this RBH4MNH program overlap with any GIZ programs?
If so, how do they overlap? Are you able to share information between the programs?
Do the programs work together? If so, how? Why or why not?

7. Observing the program from your angle, what would you describe as the main challenges its implementation faced?
Could you do anything, as GiZ staff, to assist?

8. How, in your opinion, has been the general response of (i) the Ministry of Health and (ii) the government to the RBF4MNH program?
Probe: Response of RHU?
What was the level of comfort with this program?
Did they appear agreeable to this program?
Do you know of objections to this program?

9. How, in your opinion, has been the general response of other development partners to the RBF4MNH program?
Were they agreeable to the program?
Do you know of any objections?
How has this program affected the standing of OPTIONS or KfW/RNE locally?

9. How, in your opinion, has been the general response of (i)district health officers and (ii) providers to the RBF4MNH program?

Were there any differences across districts? Facilities?

Do you know of any reasons for these differences?

10. How, in your opinion, has been the general response of the local population in Malawi?

Were they receptive to the idea of this program?

Probe: Why or why not?

11. What is your overall impression of the RBF4MNH program during this initial phase?

Have you witnessed any successes?

Probes: Do you have any knowledge of how these successes were achieved?

Did you see the program facing challenges?

Probes: By whom? Do you know how the program handled these challenges?

12. In your opinion, how has the RBF4MNH program affected accessed to care and maternal health?

Probe: Improved access to care? Too early to see any changes?

Do you feel the RBF4MNH program will have an impact in these areas of health?

Probe: Why or why not?

13. What recommendations would you make in regards to the RBF4MNH program?

14. Is there anything further that you would like to add about the topics that we have spoken about today?
